# Exploratory Genomic Marker Analysis of Virulence Patterns in *Listeria monocytogenes* Human and Food Isolates

**DOI:** 10.3390/foods14101669

**Published:** 2025-05-09

**Authors:** Valeria Russini, Maria Laura De Marchis, Cinzia Sampieri, Cinzia Onorati, Piero Zucchitta, Paola De Santis, Bianca Maria Varcasia, Laura De Santis, Alexandra Chiaverini, Antonietta Gattuso, Annarita Vestri, Laura Gasperetti, Roberto Condoleo, Luigi Palla, Teresa Bossù

**Affiliations:** 1 UOC Food Microbiology Unit, Istituto Zooprofilattico Sperimentale del Lazio e della Toscana “M. Aleandri”, 00178 Rome, Italypaola.desantis@izslt.it (P.D.S.); biancamaria.varcasia@izslt.it (B.M.V.); roberto.condoleo@izslt.it (R.C.); teresa.bossu@izslt.it (T.B.); 2 Department of Public Health and Infectious Diseases, Sapienza University of Rome, 00185 Rome, Italy; annarita.vestri@uniroma1.it (A.V.); luigi.palla@uniroma1.it (L.P.); 3 National Reference Laboratory (LNR) for *Listeria monocytogenes*, Istituto Zooprofilattico Sperimentale dell’Abruzzo e del Molise “Giuseppe Caporale” (IZSAM), 64100 Teramo, Italy; a.chiaverini@izs.it; 4 Department of Food Safety, Nutrition and Veterinary Public Health, Istituto Superiore di Sanità, 00161 Rome, Italy; antonietta.gattuso@iss.it; 5 UOT Toscana Nord, Istituto Zooprofilattico Sperimentale del Lazio e della Toscana “M. Aleandri”, 56123 Pisa, Italy; laura.gasperetti@izslt.it

**Keywords:** *Listeria monocytogenes*, virulence gene, food safety, whole-genome sequencing, genomic characterization, principal component analysis

## Abstract

*Listeria monocytogenes* causes listeriosis, a severe foodborne disease with high mortality. Contamination with it poses significant risks to food safety and public health. Notably, genetic characteristic differences exist between strains causing human infections and those found in routine food inspections. This study examined the genotypic factors influencing the pathogenicity of *L. monocytogenes*, focusing on virulence gene profiles and key integrity genes like *inlA* to explain these divergences. The dataset included 958 strains isolated from human, food, and environmental samples. Whole-genome sequencing identified virulence genes, and principal component analysis (PCA) examined 92 virulence genes and *inlA* integrity to uncover potentially pathogenic patterns. The results highlight differences in virulence characteristics between strains of different origins. The integrity of *inlA* and genes such as *inlD*, *inlG*, and *inlL* were pivotal to pathogenicity. Strains with premature stop codons (PMSCs) in *inlA*, associated with reduced virulence, accounted for a low percentage of human cases but over 30% of food isolates. Sequence types (STs) like ST121, ST580, and ST199 showed unique profiles, while ST9, dominant in food, occasionally caused human cases, posing risks to vulnerable individuals. This research highlights the complexity of the pathogenicity of *L. monocytogenes* and emphasizes the importance of genomic surveillance for effective risk assessment.

## 1. Introduction

*Listeria monocytogenes* is a Gram-positive, facultative intracellular bacterium that poses significant risks to public health and food safety. It is the causative agent of listeriosis, a severe foodborne disease associated with a high case fatality rate (19.7% in 2023) and an extremely high proportion of hospitalization (96.5% in 2023). This makes listeriosis one of the most concerning foodborne diseases in the EU, with 2952 confirmed cases [1], particularly in immunocompromised individuals, the elderly, and pregnant women [2]. The disease manifests itself in various clinical forms, ranging from gastroenteritis to invasive infections such as septicaemia and meningitis, often leading to severe complications or death in vulnerable populations [3,4].

The confirmed number of cases has been slightly increasing in the past few years in the EU, based on the notification rate. The notification rate in Italy was always lower than the European average in the last few years [1,5], except in 2022 due to a large national outbreak [6].

*L. monocytogenes* represents a major threat to the food industry and public health since it is ubiquitously present in natural and industrial environments, including soil, water, vegetation, and food production facilities. Its remarkable adaptability to extreme environmental conditions, such as low temperatures, high salinity, and acidic environments, contributes to its persistence and ability to contaminate ready-to-eat foods (RTE) and other perishable items [7]. Moreover, it has also adapted to sanitizers and disinfection agents used in food processing environments, e.g., quaternary ammonium compounds (QACs), hydrogen peroxide, peracetic acid, and sodium hypochlorite [8,9,10]. This resilience is underpinned by an array of stress response mechanisms, which are tightly regulated to ensure survival and virulence under adverse conditions. *L. monocytogenes* is able to tolerate adverse environmental conditions by forming biofilms and by developing stress-resistant mechanisms, surviving for long periods in food processing plants [11]. For these reasons, during ordinary official inspections and in foodborne disease outbreak investigations, environmental samples are collected and play a crucial role in identification (environmental samples include surfaces in contact with food, such as slicers, counters, knives, sponges, cutters, etc.), as well as food samples [12,13]. The current food safety criteria adopted in Europe (Reg EC 2073/2005) [14] regulate the presence of *L. monocytogenes* in RTE foods not intended for infant or special medical purposes very strictly, requiring its absence in 25 g of foods that constitute a favourable medium for growth or a contamination level lower than 100 cfu/g in food considered an unfavourable medium for growth.

*L. monocytogenes* species are highly heterogeneous: they can be divided into 13 serotypes [15] and four molecular serogroups (IIa, IIc, IIb, and IVb) [16] related to four main lineages (I, II, III, and IV) [17]. Serotypes 1/2a, 1/2b, 1/2c, and 4b cause most human listeriosis cases [18,19,20]. These categories can be further subdivided by sequencing a small number of housekeeping genes using multilocus sequence typing (MLST) [19] and clonal complexes (CCs). Previous studies have identified hypo- and hypervirulent CCs, with CC1, CC2, CC4, and CC6 being hypervirulent in humanized models, while CC9 and CC121, of foodborne origin, show hypovirulence in vivo [4,21,22]. From an epidemiological point of view, a more accurate identification of molecular clusters derived from associated food products, the environment, and human cases can be achieved by using whole-genome sequencing (WGS) data. In particular, core genome MLST (cgMLST)-based typing is one of the most helpful methods for the identification of epidemiological clusters [23].

Genomic studies have revealed significant intra-species diversity in *L. monocytogenes*, comprising multiple lineages and serotypes. Lineage I is often associated with human listeriosis outbreaks and Lineage II is more commonly found in environmental samples and animal reservoirs [24,25,26].

The pathogenesis of *L. monocytogenes* is governed by a set of well-characterized virulence factors [27]. Some key virulence genes, such as those encoding listeriolysin O (LLO) (gene *hly*) and internalins (genes *inlA* and *inlB*), enable the bacterium to invade host cells, escape phagosomal degradation, and spread intracellularly, allowing for the evasion of the host’s immune response [28,29,30]. The PrfA-regulated virulence network also interacts with stress response pathways, highlighting the interplay between environmental adaptation and pathogenicity. In *L. monocytogenes*, four *Listeria* pathogenicity islands have been described (LIPI-1, LIPI-2, LIPI-3, and LIPI-4) [31,32,33,34].

Internalin A (InlA) and B (InlB), encoded by the *inlAB* operon, bind to E-cadherin and Met receptors, inducing bacterial uptake through endocytosis [30,35]. Mutations in the *inlA* gene, leading to premature stop codons (PMSCs), cause the dysregulated expression of internalin and reduce the strain’s capacity to invade human epithelial cells [30,36,37,38].

The European project LiSEQ [39] has highlighted how the distribution of clonal complexes and STs isolated from food does not overlap with the distribution of isolates from human clinical cases [40]. The isolate type was unevenly distributed across the genetically diverse strains, with CCs within Lineage I strongly associated with clinical cases and those within Lineage II strongly associated with isolates from food [21,39,41,42,43]. These data highlight how the risk of listeriosis can be differentiated on a genomic basis. The combination of virulence determinants could play an important role in promoting infection.

This study aimed to analyze the reasons behind the observation that certain sequence types are not commonly found in human infections but are significantly detected during routine official food inspections by the competent authorities. The strains present in food, which is the primary source of infection, could reflect those circulating in the territory and correspond to those found in human cases. However, based on the data we had, there appeared to be a discrepancy between the two groups, which we aimed to analyze and explain through an exploratory investigation of virulence genes. In this study, we examined 958 strains of *L. monocytogenes* stored in the regional laboratories of the IZSLT and collected during 2011–2023 with the aim of identifying specific genotypic traits that were strongly linked with isolates responsible for causing listeriosis. To achieve this, a principal component analysis (PCA) and a cluster analysis were carried out on the presence/absence of 92 well-known virulence genes and an additional variable related to the integrity of the *inlA* gene. This approach aimed to uncover patterns of similarity and divergence, as well as potential associations with virulence, between strains originating from human cases and those derived from food or environmental sources.

## 2. Materials and Methods

The dataset was composed of 621 strains found and isolated from food (mainly, but not exclusively, of animal origin) and environmental matrices (named VET). The strains were collected during the routine activity of the regional laboratory of the UOC Food Microbiology department (Regional Reference Center for Pathogenic Enterobacteriaceae, or CREP) of the Istituto Zooprofilattico Sperimentale del Lazio e della Toscana “M. Aleandri” (IZSLT) during the years 2011–2023.

Moreover, the dataset included 337 strains isolated from clinical cases (named UM) during the years 2018–2023 in the Lazio and Tuscany regions, sent from private laboratories and public hospitals to the regional laboratories of the UOC Food Microbiology department (CREP and the Regional Reference Laboratory for Foodborne Pathogens of Human Origin, or LRPTAU) of the IZSLT.

The UOC Food Microbiology department at the IZSLT hosts laboratories that have historically operated as official control laboratories for food microbiological analyses [44]. The regional laboratories of the UOC Food Microbiology department of the IZSLT (CREP and LRPTAU) analyze bacterial isolates obtained through the microbiological investigation of food matrices sampled by the competent authorities of the Lazio and Tuscany regions and by producers themselves for self-inspection. Furthermore, the regional CREP and LRPTAU laboratories receive human isolates from public and private hospitals and laboratories across the entire area of authority for serological identification, monitoring, and research purposes.

### 2.1. Whole-Genome Sequencing and in Silico Analysis

The entire genomes of all the available strains were sequenced using a Next-Generation Sequencing (NGS) methodology. Genomic DNA was extracted using the automatic extraction system QIASYMPHONY (QIAGEN, Hilden, Germany) or through manual extraction using a DNA MINIPREP kit (QIAGEN, Hilden, Germany), based on the use of silica-based DNA purification technology combined with the automated handling of magnetic particles, following the manufacturer’s instructions. Libraries were prepared using Nextera XT DNA Library Prep or Illumina DNA Prep following the manufacturer’s instructions and run on a MiSeq sequencer (Illumina Inc., San Diego, CA, USA) for paired-end sequencing (2 × 300 bp or 2 × 250 bp). Raw reads are available on request.

The raw reads’ quality was assessed using Fast QC v0.11.9 [45], and low-quality sequences and adapters were trimmed using Trimmomatic v0.39 [46]. In general, the following quality filters were used: a minimum quality of Q30 to keep a base from the beginning and from the end of the read, a window size of 10 with Q20 as the average quality, and a minimum length read of 50 bp. The high-quality reads were assembled de novo into contigs using SPAdes v3.13 with the “careful” option [47]. The resulting assemblies’ quality was assessed using QUAST v5.0.2 [48], considering the following minimum quality requirements: N contigs < 200, an N50 > 30,000, and a genome length of 3 Mb ± 5% [49].

The multilocus sequence type (MLST) was deduced in silico by evaluating the composition of the seven housekeeping genes (*abcZ*, *blgA*, *cat*, *dapE*, *dat*, *ldh*, and *lhkA*) [50,51] identified using BIGSdb-Lm schemes by using the MLST technique (Github https://github.com/tseemann/mlst, accessed on 2 December 2024) [52] for all the sequenced genomes.

The BIGSdb-Lm dataset was used as a reference for the 92 virulence genes [27,53,54]. The gene search was performed using the Blast tool [55]. A presence/absence matrix (0/1) for the 92 genes was created. A binary variable related to the integrity of the *inlA* gene was also added (1 = intact, without PMSCs; 0 = not intact, with PMSCs), resulting in a total of 93 analyzed variables.

### 2.2. Statistical Analysis and Principal Component Analysis

A descriptive analysis was performed to examine the main characteristics of the dataset, focusing on the distribution of sequence types (STs) and the isolation sources.

An exploratory multivariate analysis employing principal components (PCA) was carried out using a correlation matrix, employing two different approaches to investigate patterns and variability within the dataset. In the first approach (I), the principal components were calculated using exclusively the subset of samples derived from human origins. This step ensured that the components were initially shaped by the variability inherent to human-derived samples. In the subsequent step of this approach, the entire dataset, including both human- and food-derived samples, was projected onto the main principal components previously derived. This allowed for an evaluation of how food-derived samples aligned or diverged in relation to the variability structure defined by the human samples.

In the second approach (II), PCA was performed directly on the full dataset, which included both human- and food-derived samples. This approach captured the total variability of the combined dataset.

In both approaches, the selection of significant principal components followed the Kaiser Rule, which considers components with eigenvalues ≥ 1 as meaningful contributors to the dataset’s variability and the observations from the screeplot (Elbow Rule). The score plot was displayed for a visual inspection of the non-overlapping areas of sample units projected onto the main components. These two complementary approaches provided a comprehensive framework to examine the principal components and their ability to highlight and describe separate clusters within the data. Additionally, K-means clustering was performed on the binary dataset to confirm the sample groupings suggested by principal component analysis (PCA). To account for the binary nature of the dataset, similarity metrics including the matching, Dice, Jaccard, and Sneath coefficients were employed. The number of clusters (*k*) was determined manually based on the patterns observed in the PCA results. Since the collection of human isolates by the laboratory began later, the same PCA was performed on a restricted dataset which included human and food strains isolated and collected during the same period, in particular between 2018 and 2023. All statistical analyses were conducted using STATA SE 17.

## 3. Results

**Figure 1:**
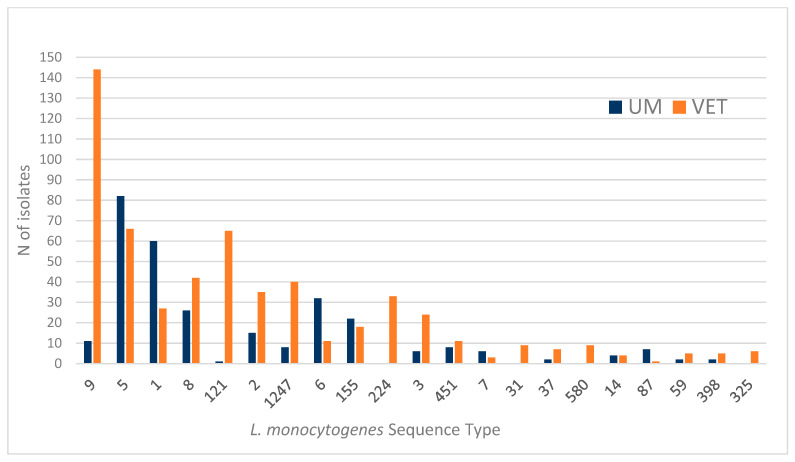
Sequence type distribution according to isolates’ origin of the samples available for this study.

All the available isolates were sequenced, meeting the minimum quality requirements necessary for robust genomic analysis. In the dataset, the frequency distributions of sequence types (STs) from strains of human origin (UM) and food and environmental origin (VET) showed limited overlap, underscoring the complexity of the mechanisms underlying human infection by *Listeria monocytogenes*. Figure 1 illustrates the distribution of STs by origin (UM or VET) for the 21 most frequently occurring STs.

For instance, ST9, which was predominantly associated with food (VET), included only 11 human cases (UM) out of 155 total isolates. Similarly, ST121 showed a marked predominance of food origins (VET), with just 1 human case out of 66 total isolates. Conversely, ST5 demonstrated a more balanced distribution, with a slightly higher frequency of human cases (82 UM vs. 66 VET). Notably, STs such as ST5, ST1, ST6, ST155, and ST87 were more frequently isolated from humans, suggesting a stronger association with human infections. On the other hand, STs such as ST9, ST8, ST121, ST2, ST1247, ST224 (only VET), ST3, ST31, ST580, and ST325 (only VET) were more commonly or exclusively associated with food or environmental samples. These findings highlight the diverse epidemiological profiles of *L. monocytogenes* across different STs and origins.

**Figure 2:**
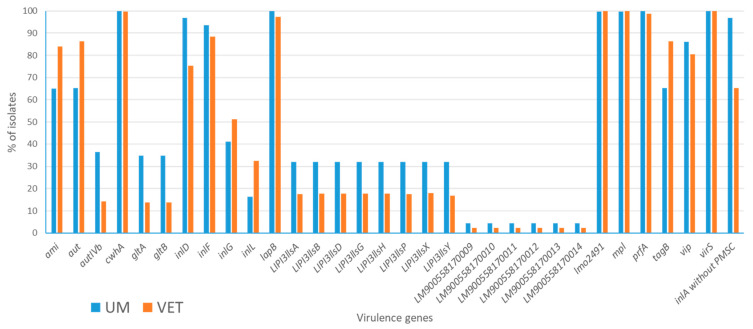
Bar chart showing the distribution of virulence genes found in isolates of human (UM) and food/environmental origin (VET).

The distribution of virulence genes across isolates is presented in Figure 2, which includes only genes that showed variability based on their isolation source (genes present or absent in 100% isolates were excluded), resulting in a total of 35 variables. A limited number of virulence genes was found to be more prevalent in veterinary isolates (VET) compared to human isolates (UM), specifically five genes: *ami*, *aut*, *inlG*, *inlL*, and *tagB*. In contrast, five genes, *cwhA*, *lmo2491*, *mpl*, *prfA*, and *virS*, demonstrated an almost identical distribution between the two sources, with presence rates exceeding 98%. The genes *inlP3*, *inlP4*, and *inlPq* were almost absent in both sources (absent in UM sources, present at 0.16% in VET sources, not reported in the figure). For the remaining 22 virulence variables, human isolates consistently exhibited a higher percentage of presence compared to veterinary isolates.

Particular attention was given to the virulence gene *inlA*, which is ubiquitous in *L. monocytogenes*. This gene is of special interest because its functionality can vary due to the presence of premature stop codons (PMSCs) that result in a truncated, non-functional form of the protein. Among human isolates, 96.7% (326 out of 337) carried a fully intact *inlA* gene without PMSCs, enabling its full functionality. In contrast, only 65.2% (405 out of 621) of veterinary isolates carried an intact *inlA* gene, with the remaining 34.8% exhibiting PMSCs. This distinction suggests a possible link between the reduced virulence of food-derived isolates and the presence of PMSCs, highlighting a potential mechanism that may influence the differential capacity of isolates to cause human infections.

In some specific sequence types (STs), the distribution of the intact *inlA* gene without premature stop codons (PMSCs) was notably consistent across human (UM) and food and environmental (VET) isolates. In ST1, ST5, ST6, and ST8, 100% of the isolates from both origins carried the intact form of *inlA*, suggesting a high potential for virulence in these groups. Conversely, the presence of PMSCs was particularly prevalent in certain STs associated predominantly with food origins. For example, among the isolates belonging to ST9, only 9% carried an intact *inlA* gene without PMSCs, and all these cases were of veterinary origin. Similarly, in ST121, only 2% of the isolates possessed an intact *inlA* gene without PMSCs. Some specific STs (almost) exclusively had isolates with PMSCs, namely ST9 (91%), ST31 (100%), ST121 (98%), ST193 (100%), ST199 (100%), and ST580 (100%).

### 3.1. Principal Component Analysis

#### 3.1.1. Results for First PCA Approach

In approach I to the principal component analysis, from the original dataset of 93 variables, a subset of 28 was selected after excluding those with zero variance (they were therefore constants, which do not contribute to variability).

The principal component analysis (PCA) revealed seven orthogonal components (PCs) with eigenvalues greater than 1, collectively explaining approximately 96% of the total variability in the dataset (Table S1). However, the first four principal components (PCs) explained most of the variance in the dataset (83.7%), effectively summarizing the distinct patterns of gene presence/absence. In particular, the first principal component (PC1) had an eigenvalue of 13.2, accounting for 47.1% of the total variance. This component was strongly influenced by a high number of variables, such as *ami*, *aut*, *autIVb*, *gltA*, *gltB*, *tagB*, and genes in the *LIPI3*-*lls* family (e.g., LIPI3-llsA, *LIPI3-llsB*) (coefficients greater than 0.25). The second principal component (PC2) had an eigenvalue of 6.04, representing 21.6% of the total variance. It was characterized by strong positive coefficients for genes in the *LIPI4* family (>0.4). The third principal component (PC3), with an eigenvalue of 2.20 (7.8% of the total variance), showed a linear contrast of *inlD* and *inlA* without PMSCs (positive coefficients), versus *inlG* and *inlL*. The fourth component had an eigenvalue of 2 (7.1% of the total variance) and it was mainly characterized by strong positive coefficients for the genes *lmo2491* and *mpl*.

In the score plots comparing PC3 against PC1, PC2, and PC4, distinct regions of non-overlap were observed between samples of food origin (VET) and human origin (UM), suggesting differences in virulence characteristics between these groups. PC3 emerged as being particularly relevant, highlighting variables linked to the integrity of internalin genes, which are associated with invasiveness. It showed a linear contrast between the presence of *inlA* without PMSCs (eigenvector coefficient = 0.55) and *inlD* (eigenvector coefficient = 0.56) with a strong positive influence and the presence of *inlG* and *inlL* (eigenvector coefficients = −0.29 and −0.44, respectively) with a strong negative influence (Table 1).

**Figure 3:**
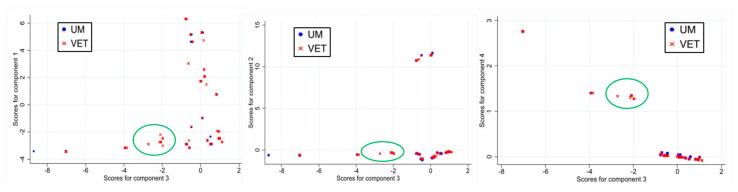
Score plot of PC3 vs. PC1, PC2, and PC4 for approach I. The area of non-overlap between samples of different origins is circled (UM: human origin; VET: food origin).

Figure 3 displays the score plots and the highlighted area of non-overlap revealed by PC3, including 75 samples (74 VET and only 1 UM), plotted against PC1, PC2, and PC4. The area circled in the score plot corresponds to samples associated with specific sequence types (STs). Notably, these included ST121 (predominantly VET samples, with only 1 UM sample out of 66), ST580 (exclusive to VET samples), and ST199 (exclusive to VET samples). In general, samples with a PC3 score below −1.90 (242 in total) belonged to one of ST9, ST31 (exclusive to VET samples), ST121, ST193, ST199, or ST580. Of these 242 samples, only 13 originated from UM samples, and only 15 had an intact sequence of the *inlA* gene, underscoring a strong association between the PC3 score and specific sequence types with a VET origin. The presence of *inlG* and *inlL* and the absence of *inlD* and *inlA* without PMSCs (or rather the presence of non-intact *inlA*) thus characterized these samples with negative scores.

The results of the cluster analyses demonstrate a consistent pattern across results obtained using different methods. Specifically, K-means clustering with *K* = 12 when using the Dice measure and *K* = 15 when using either the Sneath and Jaccard measures consistently divided the group identified by PC3 into two distinct subgroups. This division reliably included 73 of the 75 samples originally considered in the PCA, highlighting an agreement between the PCA grouping and the clustering results obtained using these methodologies.

#### 3.1.2. Results for Second PCA Approach

In approach II, from the original dataset of 93 variables, a subset of 35 was selected after excluding those with zero variance. PCA identified nine components (PCs) with eigenvalues greater than 1, collectively accounting for approximately 92% of the total explained variability (Table S2).

The first five principal components (PCs) collectively explained 74.1% of the variance in the dataset, capturing key patterns of biological significance. PC1 had an eigenvalue of 12.1 (34.6% variance), and it was dominated by positive coefficients for all the *LIPI3-lls* genes (e.g., llsA, *llsB*, *llsD*), suggesting a broad influence of these genes, while PC2 (16.7%) highlighted strong positive contributions from the *LIPI4* gene group. PC3 (8.6%) was defined by large positive coefficients for *inlP3*, *inlP4*, and *inlPq*, whereas PC4 (7.5%) showed a contrast between genes like *ami* and *aut* with high positive weights and *autIVb*, with a substantial negative coefficient (−0.29). Finally, PC5 (6.7%) featured positive coefficients for *inlL* (0.49) *inlG* (0.31), and *vip* (0.27) but also reflected variability through moderate negative contributions from other genes such as *inlD* (−0.54) and *inlA* without PMSCs (−0.36) (Table S3).

The score plots of the major components (Figure S1) in approach II did not reveal patterns of separation between strains of different origins as neatly as in the first approach. An area with no overlap can be found in the PC1 vs. PC5 score plot for a PC5 score < −1.7 (18 samples, all of VET origin). In this case, samples with a negative PC5 score were influenced by the absence of the virulence genes *inlG*, *inlL*, and *vip* and the presence of *inlA* without PMSCs and *inlD*. In the score plot for PC1 vs. PC3, an area can be found including the other 72 samples (of which 7 were UM isolates), with a positive PC1 score, characterized by the presence of *LIPI3-lls* genes, and values of PC3 < 0.39, characterized by the absence of internalin genes (*inlP3*, *inlP4*, *inlPq*).

Both groups identified through PCA were consistently present in the clustering results of the K-means analysis when using the Sneath metric with *K* = 11, comprising, clusters of, respectively, 16 samples out of 18 (all VET samples) and 72 samples (65 VET and 7 UM samples). Furthermore, the group of 72 samples emerged as a distinct cluster in almost all the clustering results for *K* > 7, regardless of the metrics employed. This consistency underscores the robustness of the clustering approach in capturing the structure suggested by PCA across different parameter settings.

#### 3.1.3. Focus on 2018–2023 Dataset

A sub-analysis was carried out using the same methods described but only considering the dataset from 2018 to 2023, excluding the years where only food-origin isolates were collected.

**Figure 4:**
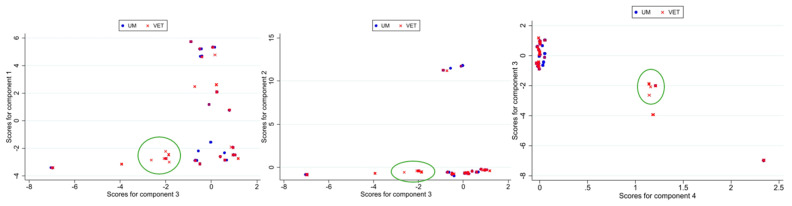
Score plot of PC3 vs. PC1, PC2, and PC4 for approach I for the 2018–2023 subset. The area of non-overlap between samples of different origins is circled (UM: human origin; VET: food origin).

The results of the first approach were very similar to the results described previously for the entire dataset. The same genes influenced the components, with similar coefficients (Table S4). From the original dataset of 93 variables, a subset of 28 (non-constant) was selected, and seven components (PCs) with eigenvalues greater than 1 collectively explained approximately 96% of the total variability in the dataset (Table S5). The first three principal components (PCs) from the binary presence–absence gene matrix explained most (76.6%) of the variance, highlighting distinct genetic patterns. PC1 (46.8%) was driven by positive contributions from the genes *gltA* and *gltB* and those in the *LIPI3-lls* gene family (e.g., llsA, *llsB*, *llsD*, *llsH*; >0.26), contrasting with negative contributions from *ami*, *aut*, and *tagB* (<−0.25). PC2 (21.8%) showed a strong positive influence from the *LIPI4* gene family (coefficients = 0.40). PC3 (8%) was dominated by positive coefficients for *inlD* (=0.57) and *inlA* without PMSCs (=0.56) and negatively influenced by *inlL* (−0.41) and *inlG* (−0.28). Even in this analysis, PC3 was able to separate a group of veterinary isolates, as shown in the score plot (Figure 4). There were 141 samples with scores < −1.8 (130 VET and 11 UM samples), which were characterized mainly by the absence of *inlD* and *inlA* without PMSCs and the presence of *inlL* and *inlG*. The non-overlapping area included 46 samples (only 1 UM isolate was present). The cluster analysis confirmed the presence of the group identified by the PCA in the K-means clustering when using the matching method with *k* = 12.

In approach II applied to the 2018–2023 dataset, from the original set of 93 variables, a subset of 31 was selected, resulting in seven components (PCs) with eigenvalues greater than 1, collectively accounting for approximately 91% of the total variability (Table S6). In this case, the eigenvector of the variables in the first seven components were different from those in approach II described before (Table S7). PC1 had an eigenvalue of 12.5, explaining 40.3% of the variance. It was strongly influenced by the *gltA*, *gltB*, and *LIPI3* genes, which exhibited high positive coefficients, contrasting with genes like *aut* and *tagB* with strong negative coefficients. PC2 (eigenvalue = 6.039, explaining 19.48% of the total variance) was characterized by high positive coefficients for *LIPI4* genes. PC3 (eigenvalue = 2.4, explaining 7.6% of the total variability) displayed high negative coefficients for *inlD* and *inlA* without PMSCs and positive ones for *inlG*, *inlL*, *lapB*, *prfA*, and *vip*, reflecting a contrast between the presence of internalin family genes and regulatory factors.

The results appeared to be more similar to those from approach I, with PC3 highlighting an area in the score plot with no overlap between UM and VET isolates (Figure S2) but with a very low number of samples (only 13 VET isolates) and low biological significance. The cluster analysis also identified the group indicated by the PCA.

## 4. Discussion

This study investigated the virulence patterns of *Listeria monocytogenes* strains originating from clinical cases and food and environmental sources. These isolates were collected in central Italy (Lazio and Tuscany regions) as part of official inspections and the institutional mandate of the CREP and LRPTAU laboratories established at the IZSLT. The dataset contained all isolates received and sequenced in the years 2011–2023. The findings highlight some critical differences in virulence characteristics between strains from different sources and provide insights into their implications for public health.

Over 30% of food isolates, especially those belonging to ST9, showed potential limited infectivity due to the presence of premature stop codons (PMSCs) in the *inlA* gene [30]. This suggests that a significant proportion of food-associated strains may not pose a substantial risk to human health under normal conditions. As a matter of fact, our analysis revealed a low percentage of human cases involving strains with PMSCs [30]. These individuals were probably particularly frail and may have encountered strains that can overcome host defences despite their reduced virulence profiles. The dissemination of good hygiene practices and conscious consumption of food is essential to reduce the exposure of these at-risk categories.

This study identified a group of *L. monocytogenes* strains from food sources with unique virulence characteristics, specifically those belonging to sequence types (STs) ST121, ST580, and ST199. The profiles of these were not fully comparable to those observed in human isolates, as revealed by principal component analysis (PCA), probably reflecting adaptation to different ecological niches and potential hosts.

PCA highlighted the critical role of several genes related to host cell adhesion and invasion, including *inlD*, *inlG*, and inlL, and the integrity of the *inlA* gene. In particular, in the first approach, the isolates with negative scores (<−1.80), mainly VET isolates, were characterized by the presence of *inlG* and *inlL* and the absence of *inlD* and *inlA* without PMSCs. This last variable in particular shows agreement with what is known and present in the literature [30,36,37,38]. In addition to confirming the importance of considering genomic features in risk assessment, this analysis reinforced the use of sequence type (ST) identification as a robust approach to summarize virulence patterns. Distinct sequence types can serve as proxies for specific genetic and functional attributes, making them valuable tools for epidemiological monitoring and risk assessment. The second approach to the PCA did not seem to identify the patterns distinguishing UM strains from VET strains as effectively as the first approach. The PCA and clustering analyses conducted on the dataset of the most recent samples (2018–2023) essentially confirmed the observations previously made in the analyses of the entire dataset. Future investigations could involve evaluating alleles with respect to the variation in the degree of virulence over time and not only the presence or absence of genes.

The clustering patterns identified by PCA were consistent with the groupings found using the clustering methods, further validating the initial observations. The consistency of results indicates that the clusters identified are meaningful and reflect the underlying structure of the data.

Across multiple studies, the presence or absence of key genes has been linked to differences in pathogenicity, host specificity, and survival under stress conditions, highlighting the need for a thorough understanding of *L. monocytogenes* as a pathogen. The internalin family, including *inlA*, *inlB*, *inlC*, and others, is essential for host cell invasion and tissue-specific infection. For example, *inlF* encodes a protein mediating brain invasion through interaction with vimentin, and its absence may attenuate virulence [3,56]. Similarly, *inlC* is crucial for liver infection and cell-to-cell spread [24,57]. Strains with *inlD* have high invasive potential, the intensity of which depends on the specific serotype [24,58]. Notably, additional internalins like *inlG* and *inlL* have been associated with increased invasiveness in vitro, although this effect may be modulated by the presence of PMSC mutations in other genes like *inlA* [4].

The presence of PMSC mutations in the *inlA* gene, leading to truncated, non-functional Internalin A, impairs host cell invasion and reduces the virulence of *L. monocytogenes* [3,4,30]. Interestingly, *inlA* PMSCs have mostly been observed in *L. monocytogenes* Lineage II strains (e.g., ST 7, 8, 9, 14, 31, 37, 121, 155, 398, 451, 580, 1247), particularly those associated with food sources, and, to a lesser extent, in Lineage I (e.g., ST1, 2, 3, 5, 6, 59, 87, 224) or clinical isolates [4,30,36]. *LIPI-3*, encoding Listeriolysin S (LLS), has been strongly associated with severe disease and epidemic strains [3,59,60,61]. LLS contributes to host gut microbiota modulation and bactericidal activity, which reinforces its role in the infection cycle of *L. monocytogenes*. Meanwhile, stress survival is mediated by clusters like SSI-1 and SSI-2, which show lineage-specific distributions, reflecting adaptive responses to environmental pressures [25]. SSI-1, for instance, is found in both Lineages I and II, while SSI-2 is exclusive to Lineage II CC121 (ST121) [25].

The presence of these virulence determinants in strains isolated from the fresh produce supply chain underscores the potential risk of *L. monocytogenes* contamination in food products [3]. Strains exhibiting genetic features commonly found in epidemic strains, such as a full-length *inlA*, *LIPI-3*, or additional internalins, warrant heightened surveillance. The identification of antimicrobial resistance genes like *fosX*, along with the efflux transporters *bcrB* and *bcrC*, reinforces the importance of monitoring genetic resistance traits to prevent outbreaks [3].

These findings collectively demonstrate that the pathogenicity of *L. monocytogenes* is shaped by a combination of a strain’s lineage, serotype, and environmental origin. The interplay between virulence factors, such as internalins, pathogenicity islands, and stress response genes highlights the genetic diversity of this pathogen and its ability to adapt to various niches.

Given the stringent criteria of the regulations currently adopted in Europe (Reg EC 2073/2005) [14], there could also be the possibility of stratifying the level of pathogenicity of *L. monocytogenes* based on the factors evaluated here. This study may represent a further step towards conducting a risk analysis aimed at detecting more precisely the strains of *L. monocytogenes* that are actually pathogenic for humans. Updating regulations relying on risk analyses also based on genomic data could benefit food business operators (FBOs) and food supply chains without affecting food safety and consumers’ health.

This study presents some limitations. The isolates from the clinical cases in the dataset were collected during a more limited time frame (2018–2023) in comparison to the food isolates. We tried to overcome this issue by analyzing the subgroup of strains isolated in the same years. From the sub-analysis, the results substantially confirmed the observations previously made in the analyses of the entire dataset. Furthermore, the study mainly included food isolates found in official inspections and therefore found randomly in the territory and not related to foodborne disease investigations. An increase in the search for strains in the territory specifically related to clinical cases would likely lead to a greater balance in the distribution of strains. Finally, the presence of a greater number of foodborne strains compared to human ones could also have created an imbalance in the total variability due to human and food sample origin using the second approach and hence cause differences in the results between the first and second approach which we somehow overcame by restricting the analysis to 2018–2023 dataset.

Advances in analytical methods, such as machine learning, offer new opportunities to study virulence at the sub-species level. A recent study [62] developed a high-performing machine learning model to predict the clinical frequency (i.e., virulence potential) of *L. monocytogenes* based on sub-species characteristics, such as their MLST or CCs. The results showed that the models could identify relationships between genomic features and the clinical frequency. Notably, the model’s performance improved when using pan-genome data compared to that of models trained on known SNPs or virulence genes, some of which have shown poor predictive power. These results suggest that pan-genome data better capture the complex patterns linking genomic features (including the presence of resistance and persistence genes) to virulence. This observation highlights the importance of understanding pathogens’ adaptations, which contribute to their ability to infect and survive in hosts.

## 5. Conclusions

This study highlights the complexity of the virulence patterns of *L. monocytogenes* from different sources. The identification of distinct profiles for foodborne/veterinary and human-associated strains highlights the need for targeted surveillance strategies. Furthermore, genetic information captured by PCA offers potential markers to assess public health risks. Future research should aim to fill gaps in the understanding of transmission dynamics, especially for strains affecting vulnerable populations. It will be necessary to continue to explore the pathogen’s virulence and interaction with external factors. The whole-genome sequencing of *L. monocytogenes* remains paramount, but synthetic descriptors such as ST identification may offer rapid tools to aid risk assessment related to food consumption.

## Figures and Tables

**Table 1:** Eigenvectors of the variables in the first 7 components (PCs) in approach I. Coefficients >0.25 and <−0.25 are in red.

Variable	Comp1	Comp2	Comp3	Comp4	Comp5	Comp6	Comp7
***ami***	0.2554	0.0232	0.0064	−0.0002	0.051	0.2571	0.1926
***aut***	0.2601	0.0236	0.012	−0.0006	0.05	0.2506	0.1836
***autIVb***	0.2518	0.0612	−0.0088	0.0001	−0.0588	−0.2233	−0.2093
***gltA***	0.2601	−0.0236	−0.012	0.0006	−0.05	−0.2506	-0.1836
***gltB***	0.2601	−0.0236	−0.012	0.0006	−0.05	−0.2506	−0.1836
***inlD***	0.0474	0.0188	0.5649	−0.2424	0.1889	−0.044	0.0657
***inlF***	0.0005	−0.0004	−0.0133	−0.0125	0.0437	0.5997	−0.7838
***inlG***	0.0675	−0.0693	−0.2914	0.0161	0.6334	−0.1671	−0.0459
***inlL***	0.1021	−0.0349	−0.4174	0.026	0.1763	−0.1009	0.0537
***LIPI3-llsA***	0.266	−0.0374	−0.044	0.0029	0.0797	0.1654	0.1291
***LIPI3-llsB***	0.266	−0.0374	−0.044	0.0029	0.0797	0.1654	0.1291
***LIPI3-llsD***	0.266	−0.0374	−0.044	0.0029	0.0797	0.1654	0.1291
***LIPI3-llsG***	0.266	−0.0374	−0.044	0.0029	0.0797	0.1654	0.1291
***LIPI3-llsH***	0.266	−0.0374	−0.044	0.0029	0.0797	0.1654	0.1291
***LIPI3-llsP***	0.266	−0.0374	−0.044	0.0029	0.0797	0.1654	0.1291
***LIPI3-llsX***	0.266	−0.0374	−0.044	0.0029	0.0797	0.1654	0.1291
***LIPI3-llsY***	0.266	−0.0374	−0.044	0.0029	0.0797	0.1654	0.1291
***LIPI4-LM900558170009***	0.0339	0.4031	−0.029	0.0017	0.0337	0.0068	0.0091
***LIPI4-LM900558170010***	0.0339	0.4031	−0.029	0.0017	0.0337	0.0068	0.0091
***LIPI4-LM900558170011***	0.0339	0.4031	−0.029	0.0017	0.0337	0.0068	0.0091
***LIPI4-LM900558170012***	0.0339	0.4031	−0.029	0.0017	0.0337	0.0068	0.0091
***LIPI4-LM900558170013***	0.0339	0.4031	−0.029	0.0017	0.0337	0.0068	0.0091
***LIPI4-LM900558170014***	0.0339	0.4031	−0.029	0.0017	0.0337	0.0068	0.0091
***lmo2491***	0.0141	0.0057	0.2163	0.6639	0.0644	−0.0034	0.0029
***mpl***	0.0141	0.0057	0.2163	0.6639	0.0644	−0.0034	0.0029
***tagB***	0.2601	0.0236	0.012	−0.0006	0.05	0.2506	0.1836
***vip***	0.0891	0.0284	−0.0581	0.0067	−0.6343	0.1177	0.1636
***inlA*** **without PMSCs**	0.0469	0.0181	0.5508	−0.2419	0.2141	−0.0109	0.0087

## Data Availability

The original contributions presented in the study are included in the article/Supplementary Material, further inquiries can be directed to the corresponding authors.

## Supplementary Materials

The following supporting information can be downloaded at https://www.mdpi.com/article/10.3390/foods14101669/s1: Figure S1: Score plot of PC1 vs. PC2, PC3, PC4, and PC5 for approach II. The area of non-overlap between samples of different origins is highlighted (UM: human origin; VET: food origin). Figure S2: Score plot of PC3 vs. PC1, PC2, and PC4 for approach II for the 2018–2023 subset. The area of non-overlapping between samples of different origins is highlighted (UM: human origin; VET: food origin). Table S1: Eigenvalues of the 12 components (PCs) found in approach I. Table S2: Eigenvalues of the 21 components found in approach II. Table S3: Eigenvectors of the variables in the first 9 components in approach II. Table S4: Eigenvectors of the variables in the first 7 components in approach I for the 2018–2023 subset. Table S5: Eigenvalues of the 12 components found in approach I for the 2018–2023 subset. Table S6: Eigenvalues of the first 12 components found in approach II for the 2018–2023 subset. Table S7: Eigenvectors of the variables in the first 7 components in approach II for the 2018–2023 subset.

## Abbreviations

The following abbreviations are used in this manuscript:

IZSLT	Istituto Zooprofilattico Sperimentale del Lazio e della Toscana “M. Aleandri”
UOC	Unità Operativa Complessa—Complex Operational Unit
UOT	Unità Operativa Territoriale—Territorial Operational Unit
CREP	Regional Reference Center for Pathogenic Enterobacteriaceae
LRPTAU	Regional Reference Laboratory for Foodborne Pathogens of Human Origin
PMSC	Premature stop codon
