# Supplementary material for: Exploratory Genomic Marker Analysis of Virulence Patterns in Listeria monocytogenes Human and Food Isolates
Source: Foods. 2025 May 9;14(10):1669. doi: 10.3390/foods14101669 (PMC12110734; doi:10.3390/foods14101669)
Supplement: Supplementary file 1 [file foods-14-01669-s001.zip › foods-3570891-supplementary.pdf]

Supplementary Materials

Table S1. Eigenvalue of the 12 component (PCs) found in the approach I

Component	Eigenvalue	Difference	Cumulative
**Comp1**	13.1975	7.15742	0.4713
**Comp2**	6.04007	3.84104	0.6871
**Comp3**	2.19904	0.19643	0.7656
**Comp4**	2.00261	0.622424	0.8371
**Comp5**	1.38018	0.370651	0.8864
**Comp6**	1.00953	0.0019768	0.9225
**Comp7**	1.00755	0.279236	0.9584
**Comp8**	0.728318	0.46212	0.9845
**Comp9**	0.266198	0.176471	0.994
**Comp10**	0.0897265	0.0387794	0.9972
**Comp11**	0.0509472	0.0226053	0.999
**Comp12**	0.0283418	0.0283418	1

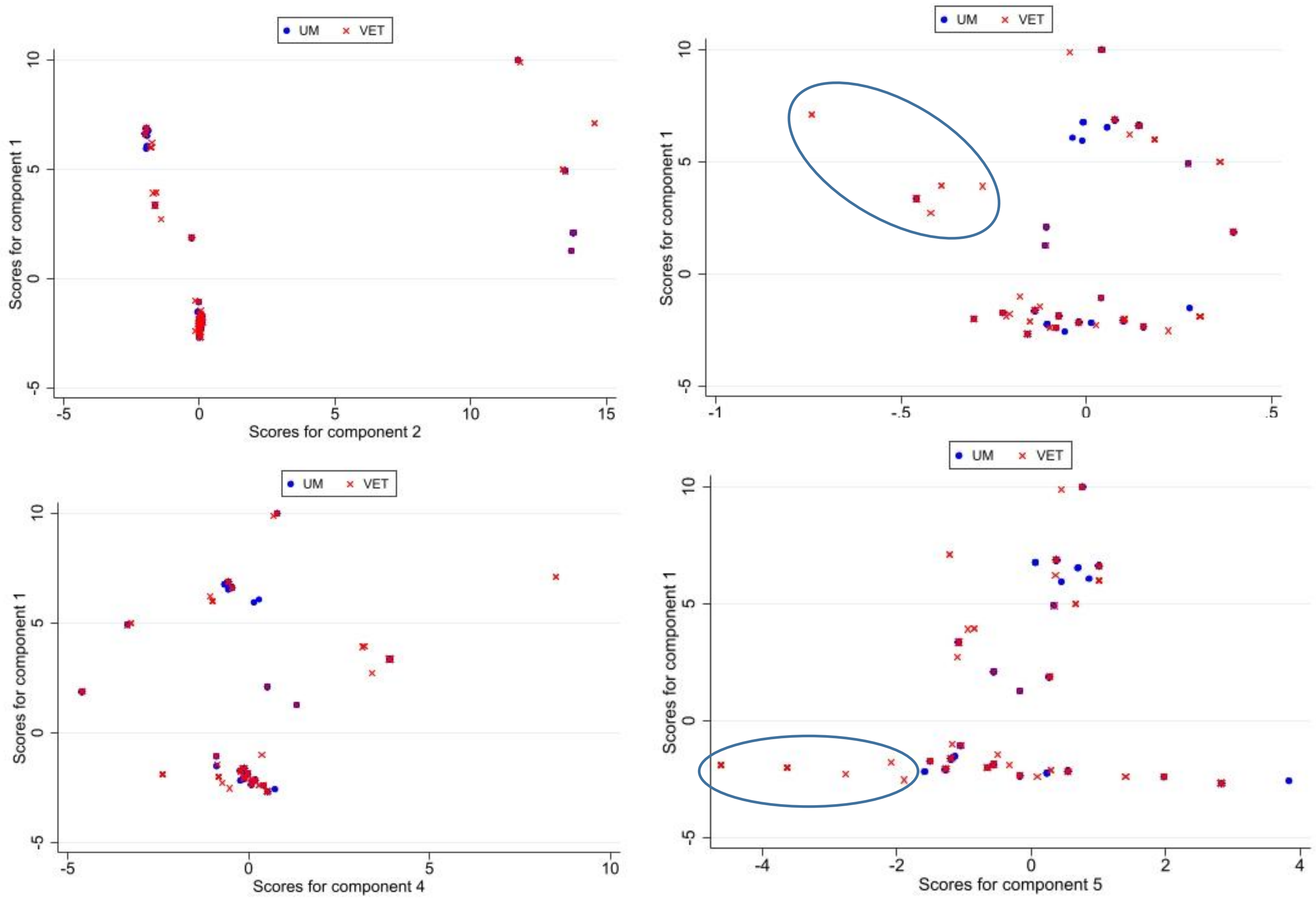

Figure S1. Score plot PC1 vs. PC2, PC3, PC4 and PC5 for the approach II. The area of non-overlapping between samples of different origins is highlighted (UM: human origin; VET: food origin).

Table S2. Eigenvalue of the 21 component found in the approach II

Component	Eigenvalue	Difference	Proportion	Cumulative
**Comp1**	12.1092	6.27011	0.346	0.346
**Comp2**	5.83905	2.81701	0.1668	0.5128
**Comp3**	3.02204	0.389444	0.0863	0.5992
**Comp4**	2.6326	0.297997	0.0752	0.6744
**Comp5**	2.3346	0.329448	0.0667	0.7411
**Comp6**	2.00515	0.235975	0.0573	0.7984
**Comp7**	1.76918	0.217196	0.0505	0.8489
**Comp8**	1.55198	0.54711	0.0443	0.8933
**Comp9**	1.00487	0.115852	0.0287	0.922
**Comp10**	0.889018	0.0896923	0.0254	0.9474
**Comp11**	0.799325	0.454359	0.0228	0.9702
**Comp12**	0.344967	0.0183441	0.0099	0.9801
**Comp13**	0.326622	0.175707	0.0093	0.9894
**Comp14**	0.150916	0.0498231	0.0043	0.9937
**Comp15**	0.101093	0.0530889	0.0029	0.9966
**Comp16**	0.0480038	0.016368	0.0014	0.998
**Comp17**	0.0316359	0.0069756	0.0009	0.9989
**Comp18**	0.0246603	0.018733	0.0007	0.9996
**Comp19**	0.00592727	4.648E-05	0.0002	0.9997
**Comp20**	0.00588079	0.0025575	0.0002	0.9999
**Comp21**	0.0033233	0.0033233	0.0001	1

Table S3. Eigenvector of the variable in the first 9 component in the approach II. Coefficients >0.25 and <-0.25 are highlighted in red.

Variable	Comp1	Comp2	Comp3	Comp4	Comp5	Comp6	Comp7	Comp8	Comp9
ami	0.23	0.0296	-0.075	0.3155	-0.0593	-0.0054	0.1208	0.0256	0.0045
aut	0.2417	0.0302	-0.0296	0.3075	-0.1097	-0.0004	-0.0107	-0.0207	-0.003
autIVb	0.2418	0.0173	0.0278	-0.2927	0.1008	-0.0002	0.0146	0.0234	-0.0002
cwhA	0.0268	-0.1142	0.0112	-0.1478	0.0239	-0.0006	0.0292	-0.0071	0.0157
gltA	0.2417	-0.0302	0.0296	-0.3075	0.1097	0.0004	0.0107	0.0207	0.003
gltB	0.2417	-0.0302	0.0296	-0.3075	0.1097	0.0004	0.0107	0.0207	0.003
inlD	0.0997	-0.0007	0.0228	-0.0891	-0.5372	-0.0795	0.1794	0.0531	0.0392
inlF	0.0286	-0.0209	0.0257	0.0287	0.0936	-0.0356	-0.1718	0.5823	-0.1416
inlG	0.1167	-0.02	0.0324	0.0522	0.3153	-0.014	-0.1309	0.4588	0.0611
inlL	0.125	0.0007	0.024	0.0921	0.4909	0.009	-0.1493	0.098	-0.0238
inlP3	0.0064	-0.0008	0.5713	0.0592	0.0013	0.0038	0.0167	-0.0443	-0.0032
inlP4	0.0064	-0.0008	0.5713	0.0592	0.0013	0.0038	0.0167	-0.0443	-0.0032

inlPq	0.0064	-0.0008	0.5713	0.0592	0.0013	0.0038	0.0167	-0.0443	-0.0032
lapB	0.0222	-0.0001	-0.009	0.0786	0.2269	-0.0152	0.6121	0.1091	0.0258
LIPI3-llsA	0.2618	-0.0877	-0.0163	0.208	0.0075	0.0031	-0.0433	0.0015	0.0039
LIPI3-llsB	0.2619	-0.0881	-0.0166	0.2109	0.0064	0.0031	-0.043	0.001	0.0038
LIPI3-llsD	0.2619	-0.0881	-0.0166	0.2109	0.0064	0.0031	-0.043	0.001	0.0038
LIPI3-llsG	0.2619	-0.0881	-0.0166	0.2109	0.0064	0.0031	-0.043	0.001	0.0038
LIPI3-llsH	0.2619	-0.0881	-0.0166	0.2109	0.0064	0.0031	-0.043	0.001	0.0038
LIPI3-llsP	0.2611	-0.0875	-0.0168	0.2123	0.006	0.0031	-0.043	0.0011	0.0039
LIPI3-llsX	0.2613	-0.0881	-0.0168	0.2109	0.0051	0.003	-0.0419	0.0006	0.0035
LIPI3-llsY	0.2579	-0.085	-0.0176	0.2174	0.001	0.0032	-0.0413	-0.006	0.0027
LIPI4-LM900558170009	0.0897	0.3916	-0.001	0.0389	0.0111	0.0004	-0.0147	0.01	0.0013
LIPI4-LM900558170010	0.0897	0.3916	-0.001	0.0389	0.0111	0.0004	-0.0147	0.01	0.0013
LIPI4-LM900558170011	0.0897	0.3916	-0.001	0.0389	0.0111	0.0004	-0.0147	0.01	0.0013
LIPI4-LM900558170012	0.0897	0.3916	-0.001	0.0389	0.0111	0.0004	-0.0147	0.01	0.0013
LIPI4-LM900558170013	0.0897	0.3916	-0.001	0.0389	0.0111	0.0004	-0.0147	0.01	0.0013
LIPI4-LM900558170014	0.0897	0.3916	-0.001	0.0389	0.0111	0.0004	-0.0147	0.01	0.0013
lmo2491	0.0069	0	0.0006	-0.009	-0.0532	0.7005	0.0336	0.0627	-0.0015
mpl	0.0069	0	0.0006	-0.009	-0.0532	0.7005	0.0336	0.0627	-0.0015
prfA	0.0143	0.0004	-0.0093	0.0833	0.1816	-0.0212	0.625	0.1648	0.0331
tagB	0.2417	0.0302	-0.0296	0.3075	-0.1097	-0.0004	-0.0107	-0.0207	-0.003
vip	0.0807	0.0001	-0.0665	0.0387	0.2726	0.0245	0.2249	-0.4484	-0.0965
virS	0.0048	-0.0003	0.0022	0.0027	0.0289	0.0019	-0.0354	0.0156	0.9806
***inlA* without PMSC**	0.1126	-0.0037	0.0332	-0.048	-0.3656	-0.0958	0.2158	0.4247	-0.0301

Table S4. Eigenvectors of the variables in the first 7 components in approach I for the 2018–2023 subset. Coefficients >0.25 and <-0.25 are highlighted in red.

Variable	Comp1	Comp2	Comp3	Comp4	Comp5	Comp6	Comp7
ami	-0.2557	0.01	0.009	-0.0001	0.0483	0.3143	-0.0745
aut	-0.261	0.0099	0.0153	-0.0002	0.0478	0.3036	-0.0678
autIVb	0.2496	0.0843	-0.0145	-0.0001	-0.0544	-0.2839	0.105
gltA	0.261	-0.0099	-0.0153	0.0002	-0.0478	-0.3036	0.0678
gltB	0.261	-0.0099	-0.0153	0.0002	-0.0478	-0.3036	0.0678
inlD	0.0482	0.0256	0.5785	-0.2158	0.1786	-0.0129	-0.0743
inlF	-0.0003	-0.0017	-0.0181	-0.0135	0.041	0.2395	0.9563
inlG	-0.0603	-0.0759	-0.2838	-0.0063	0.6436	-0.1636	-0.0259
inlL	-0.1029	-0.0467	-0.4105	-0.0033	0.1789	-0.065	-0.0996
LIPI3-llsA	0.2674	-0.0264	-0.0423	-0.0001	0.0764	0.2045	-0.0532
LIPI3-llsB	0.2674	-0.0264	-0.0423	-0.0001	0.0764	0.2045	-0.0532
LIPI3-llsD	0.2674	-0.0264	-0.0423	-0.0001	0.0764	0.2045	-0.0532
LIPI3-llsG	0.2674	-0.0264	-0.0423	-0.0001	0.0764	0.2045	-0.0532
LIPI3-llsH	0.2674	-0.0264	-0.0423	-0.0001	0.0764	0.2045	-0.0532
LIPI3-llsP	0.2674	-0.0264	-0.0423	-0.0001	0.0764	0.2045	-0.0532
LIPI3-llsX	0.2674	-0.0264	-0.0423	-0.0001	0.0764	0.2045	-0.0532
LIPI3-llsY	0.2674	-0.0264	-0.0423	-0.0001	0.0764	0.2045	-0.0532
LIPI4-LM900558170009	0.0137	0.4034	-0.0326	-0.0002	0.0362	0.013	-0.0064

Variable	Comp1	Comp2	Comp3	Comp4	Comp5	Comp6	Comp7
LIPI4-LM900558170010	0.0137	0.4034	-0.0326	-0.0002	0.0362	0.013	-0.0064
LIPI4-LM900558170011	0.0137	0.4034	-0.0326	-0.0002	0.0362	0.013	-0.0064
LIPI4-LM900558170012	0.0137	0.4034	-0.0326	-0.0002	0.0362	0.013	-0.0064
LIPI4-LM900558170013	0.0137	0.4034	-0.0326	-0.0002	0.0362	0.013	-0.0064
LIPI4-LM900558170014	0.0137	0.4034	-0.0326	-0.0002	0.0362	0.013	-0.0064
lmo2491	0.0149	0.0079	0.1819	0.6731	0.0671	-0.0016	-0.0043
mpl	0.0149	0.0079	0.1819	0.6731	0.0671	-0.0016	-0.0043
tagB	-0.261	0.0099	0.0153	-0.0002	0.0478	0.3036	-0.0678
vip	0.0849	0.0358	-0.0697	0.0048	-0.6343	0.1667	-0.1066
***inlA* without PMSC**	0.0497	0.0256	0.5684	-0.2167	0.1999	-0.0051	-0.0118

Table S5. Eigenvalues of the 12 components found in approach I for the 2018–2023 subset.

Component	Eigenvalue	Difference	Proportion	Cumulative
**Comp1**	13.1147	7.0106	0.4684	0.4684
**Comp2**	6.10415	3.87177	0.218	0.6864
**Comp3**	2.23238	0.22398	0.0797	0.7661
**Comp4**	2.0084	0.649445	0.0717	0.8378
**Comp5**	1.35895	0.329921	0.0485	0.8864
**Comp6**	1.02903	0.0220217	0.0368	0.9231
**Comp7**	1.00701	0.263624	0.036	0.9591
**Comp8**	0.743388	0.469066	0.0265	0.9856
**Comp9**	0.274322	0.222407	0.0098	0.9954
**Comp10**	0.0519152	0.0069701	0.0019	0.9973
**Comp11**	0.0449451	0.0141871	0.0016	0.9989
**Comp12**	0.030758	0.030758	0.0011	1

Table S6. Eigenvalues of the first 12 components found in approach II for the 2018–2023 subset.

Component	Eigenvalue	Difference	Proportion	Cumulative
**Comp1**	12.5054	6.46638	0.4034	0.4034
**Comp2**	6.03902	3.69227	0.1948	0.5982
**Comp3**	2.34675	0.340522	0.0757	0.6739
**Comp4**	2.00622	0.044063	0.0647	0.7386
**Comp5**	1.96216	0.114149	0.0633	0.8019
**Comp6**	1.84801	0.337893	0.0596	0.8615
**Comp7**	1.51012	0.635558	0.0487	0.9102
**Comp8**	0.874559	0.058069	0.0282	0.9385
**Comp9**	0.81649	0.43334	0.0263	0.9648
**Comp10**	0.38315	0.097194	0.0124	0.9772
**Comp11**	0.285957	0.115143	0.0092	0.9864
**Comp12**	0.170814	0.061023	0.0055	0.9919

**Comp13**	0.109791	0.053422	0.0035	0.9954
**Comp14**	0.056369	0.01892	0.0018	0.9973
**Comp15**	0.037449	0.00378	0.0012	0.9985
**Comp16**	0.033669	0.025414	0.0011	0.9995
**Comp17**	0.008254	0.002424	0.0003	0.9998
**Comp18**	0.005831	0.005831	0.0002	1

Table S7. Eigenvectors of the variables in the first 7 components in approach II for the 2018–2023 subset. Coefficients >0.25 and <-0.25 are highlighted in red.

Variable	Comp1	Comp2	Comp3	Comp4	Comp5	Comp6	Comp7
ami	-0.2372	0.0431	0.0911	-0.0222	0.3245	0.0491	-0.0194
aut	-0.2506	0.043	0.0266	-0.0107	0.2971	-0.0888	-0.0462
autIVb	0.2487	0.0168	-0.0292	0.0092	-0.2885	0.0914	0.0497
cwhA	-0.0254	-0.1501	-0.0531	0.0026	-0.2002	0.0933	0.0119
gltA	0.2506	-0.043	-0.0266	0.0107	-0.2971	0.0888	0.0462
gltB	0.2506	-0.043	-0.0266	0.0107	-0.2971	0.0888	0.0462
inlD	0.0882	0.0142	-0.5173	-0.1396	0.1823	0.1618	0.0125
inlF	0.0248	-0.0161	0.0621	-0.0383	0.0179	-0.0865	0.5897
inlG	-0.0938	-0.0425	0.3179	0.0036	-0.0505	-0.0742	0.4961
inlL	-0.1122	-0.0169	0.4914	0.0448	-0.1453	-0.1154	0.1193
lapB	0.0244	0.004	0.2688	-0.0433	0.1266	0.584	0.0283
LIPI3-llsA	0.266	-0.0487	0.0851	0.0016	0.1809	-0.0923	-0.0113
LIPI3-llsB	0.266	-0.0487	0.0851	0.0016	0.1809	-0.0923	-0.0113
LIPI3-llsD	0.266	-0.0487	0.0851	0.0016	0.1809	-0.0923	-0.0113
LIPI3-llsG	0.266	-0.0487	0.0851	0.0016	0.1809	-0.0923	-0.0113
LIPI3-llsH	0.266	-0.0487	0.0851	0.0016	0.1809	-0.0923	-0.0113
LIPI3-llsP	0.2649	-0.048	0.0855	0.0016	0.1825	-0.0928	-0.0112
LIPI3-llsX	0.2651	-0.0486	0.0837	0.0013	0.1822	-0.091	-0.0124
LIPI3-llsY	0.2606	-0.0452	0.0822	0.0013	0.1902	-0.0936	-0.0229
LIPI4-LM900558170009	0.0581	0.3972	0.0194	0.0025	-0.0126	-0.0123	0.0155
LIPI4-LM900558170010	0.0581	0.3972	0.0194	0.0025	-0.0126	-0.0123	0.0155
LIPI4-LM900558170011	0.0581	0.3972	0.0194	0.0025	-0.0126	-0.0123	0.0155
LIPI4-LM900558170012	0.0581	0.3972	0.0194	0.0025	-0.0126	-0.0123	0.0155
LIPI4-LM900558170013	0.0581	0.3972	0.0194	0.0025	-0.0126	-0.0123	0.0155
LIPI4-LM900558170014	0.0581	0.3972	0.0194	0.0025	-0.0126	-0.0123	0.0155
lmo2491	0.0084	0.0014	-0.0905	0.6878	0.0727	0.0839	0.0665
mpl	0.0084	0.0014	-0.0905	0.6878	0.0727	0.0839	0.0665
prfA	0.0172	0.0042	0.2331	-0.0529	0.1581	0.5942	0.0643
tagB	-0.2506	0.043	0.0266	-0.0107	0.2971	-0.0888	-0.0462
vip	0.0811	0.0106	0.2528	0.0284	-0.0632	0.2123	-0.468
***inlA* without PMSC**	0.1003	0.0124	-0.3236	-0.156	0.1958	0.2722	0.3803

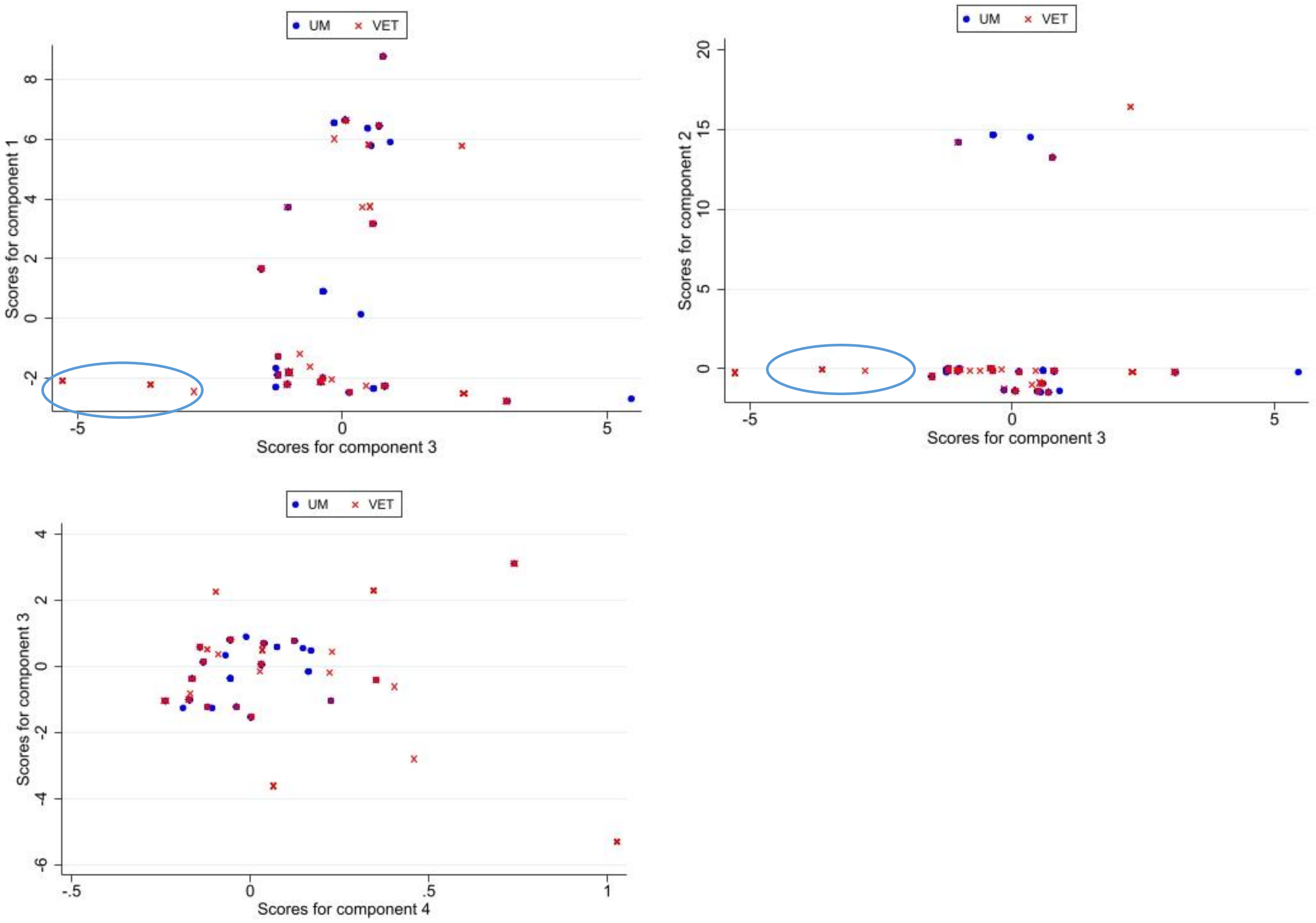

Figure S2. Score plot of PC3 vs. PC1, PC2, and PC4 for approach II for the 2018–2023 subset. The area of non-overlapping between samples of different origins is highlighted (UM: human origin; VET: food origin)
